# Erysipelas Treated with Antistreptococcus Serum

**Published:** 1902-08

**Authors:** L. Bradley Dorr

**Affiliations:** Buffalo, N. Y.


### Erysipelas Treated with Antistreptococcus Serum.

BY L. BRADLEY DORR, M. D., Buffalo, N. Y.

I WISH to report two cases of erysipelas in infants successfully treated with antistreptococcus serum. I have used the serum many times in adults with marked success, but the age of these children makes the cases interesting.

Case I.—A boy eight months old, who had erysipelas of the face and head. The edema was so great that eyes and nose were obliterated. The temperature of the surface of the body was 105° F. and the pulse 150. Five c.c. of the serum was given and a local application of ichthyol. In forty-eight hours the child was well.

Case II.—A child two weeks old, who had erysipelas over the anterior part of the body from the umbilicus to its knees. The point of infection was the penis from circumcision. The temperature of the body surface was 105.2° F., the pulse was too rapid to count and the child was convulsive. The same treatment was used as in the first case with as good and prompt result.

